# Development and performance assessment of a luminex xMAP^®^ direct hybridization assay for the detection and identification of indoor air fungal contamination

**DOI:** 10.1371/journal.pone.0173390

**Published:** 2017-03-09

**Authors:** Xavier Libert, Ann Packeu, Fabrice Bureau, Nancy H. Roosens, Sigrid C. J. De Keersmaecker

**Affiliations:** 1 Platform Biotechnology and Molecular Biology, Scientific Institute of Public Health (WIV-ISP), Brussels, Belgium; 2 Cellular and Molecular Immunology, Groupe Interdisciplinaire de Génoprotéomique Appliquée (GIGA), Université de Liège (ULg), Sart-Tilman, Belgium; 3 Mycology and Aerobiology, Scientific Institute of Public Health (WIV-ISP), Brussels, Belgium; University of Szeged, HUNGARY

## Abstract

Considered as a public health problem, indoor fungal contamination is generally monitored using classical protocols based on culturing. However, this culture dependency could influence the representativeness of the fungal population detected in an analyzed sample as this includes the dead and uncultivable fraction. Moreover, culture-based protocols are often time-consuming. In this context, molecular tools are a powerful alternative, especially those allowing multiplexing. In this study a Luminex xMAP^®^ assay was developed for the simultaneous detection of 10 fungal species which are most frequently in indoor air and that may cause health problems. This xMAP^®^ assay was found to be sensitive, i.e. its limit of detection is ranging between 0.05 and 0.01 ng of gDNA. The assay was subsequently tested with environmental air samples which were also analyzed with a classical protocol. All the species identified with the classical method were also detected with the xMAP^®^ assay, however in a shorter time frame. These results demonstrate that the Luminex xMAP^®^ fungal assay developed in this study could contribute to the improvement of public health and specifically to the indoor fungal contamination treatment.

## Introduction

Currently, indoor airborne fungal contamination is suggested to be associated with public health problems [1]. Even if indoor air fungal contaminants could be allergenic or could have an implication in respiratory diseases, such as asthma, wheezing or rhinitis [2–7] the scientific evidence for the causal link between these molds and adverse health effects is still poorly documented. The use of classical methods involving culturing and microscopic visualization in routine monitoring analysis could be pointed out as one of the reasons explaining this lack of evidence in scientific literature. Even if culture-dependent tools are useful and well documented, these techniques are known to be affected by competition factors, selection of growth media or culture conditions [8, 9] and they are only able to detect the living fungal fraction. Therefore, these technical problems could cause an underestimation of the diversity of the indoor air fungal population and reduce the evidence for the causal link between fungal contamination and health problems [8]. Another drawback is that these classical tools are time-consuming, taking 5 up to even 21 days in some cases [8, 10–12].

In order to avoid these problems of culture dependency and being time-consuming, molecular tools are increasingly being used to detect indoor airborne molds. Amongst them are the real-time polymerase chain reaction (qPCR) tools which are more and more developed for their application in fungal monitoring [13–15]. Besides being fast, accurate and specific, qPCR methods have also the advantage of being culture-independent. Furthermore, qPCR could be developed for multiplex detection, detecting simultaneously several different species, reducing once more the time and amount of sample needed for an analysis. Many qPCR assays are based on hydrolysis probes, such as the TaqMan^®^ ones, which are highly specific and useful in multiplex analysis [2, 16, 17]. However, although multiplexing is possible and already successfully performed, the number of targets is still limited to 4 or 5, especially because the number of available fluorophores and quenchers [18], which can be detected at the same time, is limited.

The Luminex xMAP^®^ technology has been demonstrated to be a valuable alternative to the qPCR multiplex. This technology is based on the detection of multiple sets of polystyrene microspheres (beads) characterized by a specific spectral emission. The number of sets that can be detected is dependent on the type of Luminex instrument being used. For the benchtop model, the MagPix instrument, 50 sets of beads can be simultaneously detected. The coupling of each set of xMAP^®^ beads with a specific oligonucleotide probe, specific to a certain target, permits to detect up to 50 different targets in a single assay [19]. One set of coupled beads-probes is hybridizing to a specific PCR amplicon previously amplified with biotinylated primers. The addition of streptavidin-R-phycoerythrin as a reporter allows the detection of each hybridized PCR amplicon on a beads set coupled with the specific probe [19]. Therefore, if the target was present in the sample, it will be detected through a green fluorescent signal read out on the Luminex instrument. Today, fungal xMAP^®^ assays have been mainly used for the diagnosis of relevant fungal pathogens in clinical samples such as e.g., some *Aspergillus* sp., *Candida* sp., *Mucor* sp. or *Fusarium* sp. [20, 21]. These assays included the testing of isolated colonies from biological samples such as biopsy’s tissues, bronchoaveolar secretions or blood. These were not yet tested on DNA extracted directly from environmental samples [20, 21]. In fact, until now, no Luminex xMAP^®^ tool is available for indoor air fungal monitoring.

In this study, a Luminex xMAP^®^ assay was developed, for the first time, for the multiplex detection, without prior cultivation, of the 10 airborne fungal species most frequently found in indoor air in Belgium [22] as well as in Europe [2], and that may cause health problems such as allergies, asthma or rhinitis [2, 4, 22]. These species are *Alternaria alternata*, *Aspergillus creber*, *Aspergillus fumigatus*, *Aspergillus sydowii*, *Aspergillus versicolor*, *Cladopsorium cladosporioides*, *Cladosporium herbarum*, *Penicillium chrysogenum*, *Stachybotrys chartarum* and *Ulocladium botrytis* [2, 22, 23]. As it is required for the performance assessment of molecular tools, the specificity and sensitivity of the developed Luminex xMAP^®^ assay was determined. Finally, the assay was tested on real-life environmental samples as a proof of concept demonstrating that the Luminex xMAP^®^ technology can be used for the monitoring of indoor air fungal contamination. The development of this Luminex xMAP^®^ assay aimed at the improvement of the framework of fungal monitoring in indoor air, which will eventually improve public health. Indeed, the simultaneous detection allowed by the multiplexing permits to reduce the time required for the analysis and for the communication of the results to the involved medical team. Moreover, the use of Luminex xMAP^®^ technology has the advantage that the sample size requirements are reduced and that no skilled mycologist is needed to perform the microscopic-based identification analysis.

## Materials and methods

### Fungal strains and DNA isolation

All the fungal species and strains used in this study are listed in Table 1. All of them were purchased from the BCCM/IHEM collection located at the Scientific Institute of Public Health in Brussels (WIV-ISP, Belgium).

**Table 1:** Fungal species and probes used in this study.

Genus	Species	Reference BCCM/IHEM^a^	Probe name	Sequence 5’ -> 3’	Length	Target	Modified from Reference
*Alternaria*	*alternata*	IHEM 4969	AaltP2.2	TGAATTATTCACCCTTGTCTTTTGCGTACT	30	ITS1	17
*Aspergillus*	*creber*	IHEM 2646	VersP1	AGACTGCATCACTCTCAGGCATGAAGTTCA	30	ITS1	17
*Aspergillus*	*sydowii*	IHEM 20347	VersP1	AGACTGCATCACTCTCAGGCATGAAGTTCA	30	ITS1	17
*Aspergillus*	*versicolor*	IHEM 18884	VersP1	AGACTGCATCACTCTCAGGCATGAAGTTCA	30	ITS1	17
*Aspergillus*	*fumigatus*	IHEM 3562	AfumP1	CCCGCCGAAGACCCCAACATGAACGCTGTT	30	ITS1	20
*Cladosporium*	*cladosporioides*	IHEM 0859	CcladP1	CCGGGATGTTCATAACCCTTTGTTGTCC	28	ITS2	17
*Cladosporium*	*herbarum*	IHEM 2268	CherbP1	CTGGTTATTCATAACCCTTTGTTGTCCGACT	31	ITS1	17
*Penicillium*	*chrysogenum*	IHEM 20859	Pchris1	GCCTGTCCGAGCGTCATTTCTGCCCTCAAGC	31	ITS2	17
*Stachybotrys*	*charatum*	IHEM 0359	StachP2	CTGCGCCCGGATCCAGGCGCCCGCCGGAGA	30	ITS1	17
*Ulocladium*	*botrytis*	IHEM 0328	UloP1	TGAATTATTCACCCGTGTCTTTTGCGTACT	30	ITS1	17

^a^ Identification number as defined by the BCCM/IHEM collection, Mycology and Aerobiology, Scientific Institute for Public Health, Juliette Wytsman street 14, 1050 Brussels, Belgium.

The culture and extraction protocol were both previously described in Libert et al. (2015) [10]. Briefly, all the strains were incubated with constant agitation in a S10 Sabouraud liquid medium (Biorad, Temse, Belgium) at 25°C during 3 to 10 days depending on the species’ culture conditions. Then, after a centrifugation of 1 min at 12 000g to eliminate all of the Sabouraud liquid, 0.25 ml of acid washed glass beads (Sigma Aldrich, Diegem, Belgium) were added to the wet sample (300 mg) transferred into cryotubes and put at -80°C during 40 minutes. After that, the samples were freeze-dried overnight with a freeze-dryer Epsilon 1-6D (Martin Christ, Osterode am Harz, Germany) and subsequently bead-beaten (1 minute, maximal speed) with a Mini bead beater (Biospec Products, OK, USA).

Finally, an adapted phenol chloroform (24:1) protocol (Ashktorab and Cohen 1992) was applied to extract DNA, which was then purified with the Qiagen CTAB genomic Tip-20 kit (Qiagen Benelux–B.V., KJ Venlo, the Netherlands) and eluted with 100μl Gibco^®^ DNase, RNase, protease free water (Life Technologies, Gent, Belgium). The purity and the amount of extracted DNA were evaluated with a Nanodrop^®^ 2000 (Thermo Scientific, Wilmington, USA).DNA integrity was verified on a 2% agarose gel.

### PCR amplification

The PCR amplifications were performed in duplex in order to amplify both the internal transcribed spacer 1 and 2 (ITS1 and ITS2) regions, using the following couples of universal primers: ITS 1 (5’-TCCGTAGGTGAACCTGCGG-3’)/ ITS2 (5’-GCTGCGTTCTTCATCGATGC-3’) and ITS3 (5’-GCATCGATGAAGAACGCAGC-3’)/ ITS4 (5’- TCCTCCGCTTATTGATATGC-3’) [24]. All primers were manufactured by Eurogentec (Liège, Belgium) and purified with HPLC. The ITS1 and ITS3 forward primers were labeled at 5’ with biotin.

Duplex PCR amplifications were performed on a Swift MaxPro and Aeris thermal cycler (Esco, Barnsley, the Netherlands). All the reactions contained 4 μl of 10 X Phusion High Fidelity PCR Buffer with 15 mM MgCl_2_ (Thermo Fisher Scientific, Erembodegem, Belgium), 0.5 μl of each primer at 0.5μM, 0.4 μl of dNTPs each at 200 μM (Thermo Fisher Scientific, Erembodegem, Belgium) and 0.2 μl of High Fidelity TaqPolymerase enzyme at 0.02 U/μl (Thermo Fisher Scientific, Erembodegem, Belgium). Finally, 11.4 μl of Gibco^®^DNase, RNase, protease free water and 5 μl of pure gDNA template (1 ng/μl) were added to obtain a final volume of 20 μl per reaction.

The PCR amplifications were carried out with following thermal cycler programme: 30 s at 98°C (initial denaturation); 35 cycles of 30 s at 98°C (denaturation), of 1 min at 55°C (annealing) and 1 min at 72°C (extension); a final extension at 72°C during 10 min and a final hold at 16°C.

### Probe selection

All probes were selected from literature (qPCR Taqman probes transferred into Luminex probes, Table 1) and adapted if needed in order to have a similar range of length. Probe quality estimation (hairpins, ΔG values…) was performed with Visual OMP version 7.8.42.0 (DNA software, Washington, USA). An *in silico* analysis of each probe was done with the Thermoblast tool from Visual OMP version 7.8.42.0 (DNA software, Washington, USA).

All probes were manufactured by Eurogentec (Liège, Belgium) and tagged with a 5’-end amino modifier C12, followed by a RP-Cartridge-Gold purification.

### Probe coupling to xMAP^®^ beads

According to the Luminex xMAP^®^ technology, each specific probe is coupled to a specific set of beads. The coupling protocol used in this study is based on the Luminex recommendations for carbodiimide coupling of amine-modified oligonucleotides to MagPlex magnetic carboxylated microspheres (beads) [25]. The final concentration of the coupled beads was 12500 beads/μl. These working stocks of coupled beads were stored in the dark at 4°C until their use.

### Coupled beads hybridization and MagPix analysis

For all Luminex analyses, every coupled bead set used was diluted with 1.5X tetramethylammonium chloride (TMAC) solution containing 5 M tetramethylammonium chloride (Sigma-Aldrich, Diegem, Belgium), 75 mM Tris (Sigma-Aldrich, Diegem, Belgium), 6 mM EDTA (Sigma-Aldrich, Diegem, Belgium), and 0.15% sarkosyl, pH 8.0 (Sigma-Aldrich, Diegem, Belgium) to arrive at a final concentration for each set of coupled beads of 76 beads /μl.

The hybridization mix contained per reaction 33 μl of a specific coupled bead set (76 beads/μl), 12 μl of Tris-EDTA buffer, pH 8 (Sigma-Aldrich, Diegem, Belgium) and 5 μl of fresh PCR amplicons. The hybridization reaction was performed on an Aeris thermal cycler (Esco, Barnsley, The Netherlands) according to the following protocol i.e., a first step at 96°C during 1 min 30 s and a second one at 58°C during 30 min. Before a third incubation step of 5 min at 58°C, 25 μl of reporter mix composed of 4 μg/ml of SAPE (Streptavidin-R-Phycoerythrin) (Life Technologies, Gent, Belgium) and 1X TMAC buffer (Sigma-Aldrich, Diegem, Belgium), were added to each sample.

Finally, all analyses were performed on a MAGPIX device (Luminex Corporation, Austin, USA) equipped with the xPONENT for MAGPIX v4.2 software (Luminex Corporation, Austin, USA). The protocol applied in all runs was performed at 58°C with a minimum of 50 beads counted for each bead region. A wash of each sample was also carried out on the machine during the analysis.

Because each bead set has a unique spectral address (distinct red color code) and each PCR amplicon hybridized to the probe bound to the beads is labeled with SAPE, the fluorescence intensity (red and green) gives information on the amount of beads per region (bead set) and on the amount of beads bound to a PCR amplicon. This last information is given by the median fluorescence intensity value (MFI) and is defined for each region (bead set).

### Data analysis and interpretation

The data analysis and the interpretation of the results were based on Wuyts et al. (2015) [26]. At the end of each run, the bead counts were checked to verify whether the bead count was homogenous for all of the coupled bead sets. If this was not the case, the run was repeated. Then, the median fluorescence intensity (MFI) of each target was used to calculate a signal-to-noise ratio (SN) with the following formula i.e.,

$${SN}_{target\;a}=\frac{{MFI}_{sample\;target\;a}}{{MFI}_{NTC\;target\;a}}$$

where SN _target *a*_ is the signal-to-noise ratio observed for the set of coupled beads selected for the detection of the target *a;* MFI _sample target *a*_ corresponds to the MFI value (collected by the xPONENT software) observed for the target in a specific sample; and MFI_NTC target *a*_ is the MFI value obtained for the NTC_target *a*_

According to Wuyts et al. (2015) [26], a result was considered as positive if SN _target *a*_ ≥ 3.00. For each SN ratio close to the limit (i.e. close to 3), a t-test (95% confidence) was performed with the SN ratio obtained for the negative control. If the difference is significant (*), the results were considered as positive. If no difference was observed with the negative control, the data were considered as negative.

### Specificity test

The specificity of each coupled beads-probes was tested with 3 different assays: a simplex analysis where each probe was tested only on its targeted species (i.e., one bead set, one species) and a multiplex analysis where each species was subjected to each coupled bead set at the same time. Finally, a mix of gDNA extracted from different species was tested.

#### Simplex analysis (DNA of 1 species, 1 set of beads)

The probe specificity was firstly tested during a simplex analysis. This test consisted of the one by one analysis of all the targeted species with their specific coupled beads–probe set. So, 10 specific hybridization mixes were made, i.e. one mix per set of beads and one mix per species, and analyzed in duplicate during 3 independent runs. All analyses were performed with 5 μl of PCR amplicon, obtained as described above. The repetitions were done with DNA template extracted from independent cultures. For each mix and run, one non-template-control PCR reaction (NTC_PCR_) i.e., gDNA replaced by water, was introduced in order to evaluate the background linked to the analysis.

#### Multiplex analysis (DNA of 1 species, multiple sets of beads)

The second step of the specificity evaluation consisted of the multiplex analysis. In this test, the hybridization mix contained every of the coupled bead sets, and this mix was tested on each species in duplicate in 3 independent runs. Every analysis was performed with 5 μl of PCR amplicon, obtained for each species with 5 ng of gDNA from pure culture, as elaborated above. The repetitions were done with DNA template extracted from independent cultures. A NTC_PCR_ was added to all runs.

#### DNA mix analysis (DNA of multiple species, multiple sets of beads)

Subsequently, a Luminex analysis was performed on the product of a PCR reaction containing a mix of gDNA extracted from different species. Nine different PCR mixes were made (Table 2). Mix 1 contained 5 ng of gDNA from all targeted species as mentioned in the Table 2 and was considered as a positive control.

**Table 2:** Composition of DNA Mixes analysis.

Species	BCCM/IHEM strain ^1^	Mix								
		1	2	3	4	5	6	7	8	9
*A*.*alternata*	3320	V	**X**	V	V	V	V	V	V	V
*A*. *versicolor* ^2^	18884	V	V	**X**	V	V	V	V	V	V
*A*. *fumigatus*	3562	V	V	V	**X**	V	V	V	V	V
*C*. *cladosporioides*	859	V	V	V	V	**X**	V	V	V	V
*C*. *herbarum*	2268	V	V	V	V	V	**X**	V	V	V
*P*. *chrysogenum*	20859	V	V	V	V	V	V	**X**	V	V
*S*. *chartarum*	359	V	V	V	V	V	V	V	**X**	V
*U*. *botrytis*	328	V	V	V	V	V	V	V	V	**X**

^1^ Identification number as defined by the BCCM/IHEM collection, Mycology and Aerobiology, Scientific Institute for Public Health, Juliette Wytsman street 14, 1050 Brussels, Belgium.

V shows the presence of the species in the mix. X indicates the absence of the species in the mix.

^2^ Only DNA from *A*. *versicolor* was added for the mixes analysis. The probe VersP1 is not specific and detects also the 2 closely related species *A*. *creber and A*. *sydowii*.

For the mixes 2 to 9, gDNA of all species targeted in the test was added in the PCR mix, except for one, whereby the missing species changed for each mix (Table 2). All analyses of mixes were done in duplicate in 3 independent runs. For every analysis, a NTC_PCR_ was added.

### Sensitivity evaluation: Limit of detection

To evaluate the sensitivity of the Luminex assay developed in this study, a serial dilution of gDNA of each targeted species was made to determine the limit of detection (LOD). Because no guidelines exist on the development and the performance assessment of molecular methods for fungal detection, the LOD estimation performed in this study is based on the workflows elaborated for the validation of molecular methods for the detection of GMO and food-pathogens. In these fields, the LOD is defined as the lowest concentration of an analyte which is detected with a probability of 95% [27, 28].

In order to estimate this LOD, 9 dilutions from 10 to 0.001 ng of gDNA of each species were amplified independently in a PCR reaction according to the above described PCR protocol. All of the PCR reactions were analyzed in duplicate in 3 independent runs.

### Proof of concept with real-life environmental samples

The environmental testing was performed on real-life air samples previously collected from contaminated houses [10]. The protocols for the sampling, the DNA extraction and microscopic determination were previously described in Libert et al. (2015) [10]. All the samples were collected in duplicate. Briefly, air samples were collected in contaminated habitats using the Coriolis μ air sampler (Bertin Techologies, Montigny-Le-Bretonneux, France) which collects contaminants into a liquid (15 ml) compatible with the classical culture-based approach as well as with molecular-based tools. A first group of samples was centrifuged during 15 min at 5000 g in order to extract the DNA using an adapted phenol-chloroform DNA extraction protocol (including bead-beating and freeze-drying steps, followed by a CTAB Tip20 purification protocol [10]). In parallel, the second group of samples was analyzed according to the culture-based protocol [10, 12] which included an incubation step of 5 days at 25°C for mesophilic and 2 days at 45°C for thermophilic fungi and a microscopic visualization for species determination.

The Luminex assay was applied on samples that were all collected in the same house in 4 different rooms (i.e. bathroom, bedroom, kitchen and living-room). The analysis was performed in duplicate with 3 independent runs according to the protocols described above. The PCR amplifications were done with 5 ng of gDNA extracted from each sample. In each run a NTC_PCR_ and 8 positive controls (i.e., gDNA extracted from pure cultures of *A*. *alternata*, *A*. *versicolor*, *A*. *fumigatus*, *C*. *cladosporioides*, *C*. *herbarum*, *P*. *chrysogenum*, *S*. *chartarum* and *U*. *botrytis*) were added. It should be noted that neither *A*. *creber* nor *A*. *sydowii* positive controls were added, because their detection was done with the same probe than that for the detection of *A*. *versicolor*.

For each SN ratio close to the limit (i.e. close to 3), a t-test (confidence 95%) was performed with the higher negative results considered as a negative control. If the difference is significant (*), the results were considered as positive. If the no difference was observed with the negative control, the data were considered as negative.

To verify that no inhibitors were present in the environmental samples extracts and to confirm that if the species would be present, it could be detected with the Luminex fungal assay, a final amount of 5 ng of gDNA from pure culture of each species not-detected during the Luminex assay and the classical monitoring were spiked into the DNA extracted from the 4 environmental samples. The spiked DNA extract comes from the same culture than that was used to prepare the DNA used as positive controls. The Luminex analysis was subsequently repeated.

## Results

### Probe selection

This Luminex assay aims at the detection of the 10 fungal species most frequently found in indoor air in Belgium and in Europe and that may cause health problems i.e., *A*. *alternata*, *A*. *creber*, *A*. *fumigatus*, *A*. *sydowii*, *A*. *versicolor*, *C*. *cladosporioides*, *C*. *herbarum*, *P*. *chrysogenum*, *S*. *chartarum* and *U*. *botrytis* [2, 22, 23]. The Luminex xMAP^®^ technology used in this study is based on the direct DNA hybridization to a specific probe coupled with a unique set of beads. Specific probes to be coupled to the beads were selected in the internal transcribed spacer (ITS) regions of the ribosomal DNA. These ribosomal regions have the advantages to be conserved and to show, at the genus level, few polymorphisms [29, 30, 31], allowing the specific detection of particular species. Another advantage is the fact that the small subunit (SSU), 5.8 S and the large subunit (LSU) from the rRNA genes flanking the ITS 1 and 2 regions, are sufficiently conserved among species to design some universal primers such as the primers ITS1 and ITS2 and ITS3 and ITS4 [24] to create the PCR amplicons to be hybridized to the probes. The probes were designed based on qPCR Taqman probes available in literature. Six probes i.e., AaltP2.2, UloP1, StachP2, AfumP1, CherbP1 and VersP1, detect amplicons in ITS 1, while Pchris1 and CcladP1 detect amplicons the ITS 2 region (Table 1). All of them are specific to their target except the probe VersP1 from the EPA [15, 17] which can also hybridize to the ITS 1 region of *A*. *creber* and *A*. *sydowii*. The probes were evaluated *in silico* and adapted if needed in order to have a similar range of length (Table 1). Indeed, the stabilization of the formation of the hybridization complex between probe and PCR amplicon is assured by the addition of TMAC which reinforces AT base-pairs [19]. Consequently, the hybridization efficiency is more influenced by the length of the probe than by the nucleotide composition [19]. According to the *in silico* testing, a consensus length for an optimal detection of each target was found to be between 29 and 31 nucleotides (Table 1).

### Specificity test

The specificity of each probe was tested in three steps i.e., a simplex analysis, a multiplex analysis and the multiplex analysis of a mix of DNA. With the simplex analysis, it was verified whether the protocol and probe can detect the amplicon from the targeted species only (i.e., one species, one probe, one bead set for detection of one species); secondly, with the multiplex analysis, it was investigated if no aspecific annealing occurred when a specific amplicon was put into a mix of probes, the specific probe included (i.e., one species, multiple sets of beads mixed for detection of one species); thirdly, with the multiplex analysis of a mix of DNA, it was verified whether no incorrect detection happened when a mix of amplicons from different species was analyzed with a mix of different beads (i.e., multiple species mixed, multiple sets of beads mixed for multiple specific detection).

#### Simplex and multiplex analysis

During these tests, the PCR amplicon of each species was detected by its own specific coupled probe-bead set in every of the 6 repetitions and this both in the simplex analysis (Table 3) as well as in the multiplex analysis (Table 4). The simplex analysis yielded average SN ratios ranging between 3.50 ±0.25 (for *A*. *sydowii*) and 22.69 ±1.54 (for *U*. *botrytis*) (Table 3). The average SN ratios obtained in the multiplex analysis ranged between 3.52 ±0.05 and 27.15 ±0.18 for *C*. *cladosporioides* and *U*. *botrytis*, respectively (Table 4). During these analyses, no false positives as well as no false negatives were observed (Tables 3 and 4), following the criteria defined for obtaining a positive result (i.e., MFI ratio ≥3.00).

**Table 3:** Simplex xMAP^®^ analysis.

	BCCM/IHEM ^a^	Number of positives ^b^							
		AaltP2.2	VersP1	AfumP1	CcladP1	CherbP1	Pchris1	StachP2	UloP1
*A*.*alternata*	3320	6/6							
*A*. *creber*	2646		6/6						
*A*. *sydowii*	20347		6/6						
*A*. *versicolor*	18884		6/6						
*A*. *fumigatus*	3562			6/6					
*C*. *cladosporioides*	859				6/6				
*C*. *herbarum*	2268					6/6			
*P*. *chrysogenum*	20859						6/6		
*S*. *chartarum*	359							6/6	
*U*. *botrytis*	328								6/6
Species	BCCM/IHEM ^a^	SN ratio ^c^^,^^d^							
		AaltP2.2	VersP1	AfumP1	CcladP1	CherbP1	Pchris1	StachP2	UloP1
*A*. *alternata*	3320	**4.15 ±1.10**	** **	** **	** **	** **	** **	** **	** **
*A*. *creber*	2646	** **	**3.63 ±0.43**	** **	** **	** **	** **	** **	** **
*A*. *sydowii*	20347	** **	**3.50 ±0.25**	** **	** **	** **	** **	** **	** **
*A*. *versicolor*	18884	** **	**4.85 ±0.52**	** **	** **	** **	** **	** **	** **
*A*. *fumigatus*	3562	** **	** **	**15.24 ±2.39**	** **	** **	** **	** **	** **
*C*. *cladosporioides*	859	** **	** **	** **	**6.86 ±0.92**	** **	** **	** **	** **
*C*. *herbarum*	2268	** **	** **	** **	** **	**7.6 ±0.66**	** **	** **	** **
*P*. *chrysogenum*	20859	** **	** **	** **	** **	** **	**5.15 ±0.81**	** **	** **
*S*. *chartarum*	359	** **	** **	** **	** **	** **	** **	**4.19 ±0.11**	** **
*U*. *botrytis*	328	** **	** **	** **	** **	** **	** **	** **	**22.69 ±1.54**

^a^ identification number as defined by the BCCM/IHEM collection, Mycology and Aerobiology, Scientific Institute for Public Health, Juliette Wytsman street 14, 1050 Brussels, Belgium.

^b^ Number of positive detections obtained during 3 independent runs done in duplicate.

^c^ SN ratio defined as the average (±standard deviation) of the ratio between the MFI values for the sample and the NTC for a specific target, obtained with 3 independents runs of independent gDNA extracts of pure cultures (5 ng of gDNA).

^d^ In bold are the values considered as positive (i.e., average SN ratio ≥3).

**Table 4:** Multiplex xMAP^®^ analysis to test the bead- probe specificity.

Species	BCCM/IHEM ^a^	NTC ^b^	Number of positives ^c^							
			AaltP2.2	versP1	AfumP1	CcladP1	CherbP1	Pchris1	StachP2	UloP1
*A*.*alternata*	3320	0/6	**6/6**	0/6	0/6	0/6	0/6	0/6	0/6	0/6
*A*. *creber*	2646	0/6	0/6	**6/6**	0/6	0/6	0/6	0/6	0/6	0/6
*A*. *sydowii*	20347	0/6	0/6	**6/6**	0/6	0/6	0/6	0/6	0/6	0/6
*A*. *versicolor*	18884	0/6	0/6	**6/6**	0/6	0/6	0/6	0/6	0/6	0/6
*A*. *fumigatus*	3562	0/6	0/6	0/6	**6/6**	0/6	0/6	0/6	0/6	0/6
*C*. *cladosporioides*	859	0/6	0/6	0/6	0/6	**6/6**	0/6	0/6	0/6	0/6
*C*. *herbarum*	2268	0/6	0/6	0/6	0/6	0/6	**6/6**	0/6	0/6	0/6
*P*. *chrysogenum*	20859	0/6	0/6	0/6	0/6	0/6	0/6	**6/6**	0/6	0/6
*S*. *chartarum*	359	0/6	0/6	0/6	0/6	0/6	0/6	0/6	**6/6**	0/6
*U*. *botrytis*	328	0/6	0/6	0/6	0/6	0/6	0/6	0/6	0/6	**6/6**
Species	BCCM/IHEM ^a^	NTC	SN ratio ^c^^,^^d^							
			AaltP2.2	versP1	AfumP1	CcladP1	CherbP1	Pchris1	StachP2	UloP1
*A*.*alternata*	3320	/	**14.06 ±1.05**	1.12 ±0.55	0.42 ±0.11	0.19 ±0.09	0.21 ±0.12	0.25 ±0.46	1.78 ±0.23	1.72 ±0.17
*A*. *creber*	2646	/	1.46 ±0.12	**3.98 ±0.37**	1.17 ±0.08	1.24 ±0.02	1.28 ±0.03	1.26 ±0.01	1.3 ±0.04	1.21 ±0.11
*A*. *sydowii*	20347	/	1.95 ±0.25	**3.87 ±0.05**	1.63 ±0.11	1.20 ±0.18	1.31 ±0.18	1.15 ±0.15	2.43 ± 0.25	1.34 ±0.15
*A*. *versicolor*	18884	/	1.54 ±0.16	**5.51 ±0.15**	1.51 ±0.10	1.16 ±0.12	1.07 ±0.13	1.1 ±0.11	2.01 ±0.19	2.43 ±0.17
*A*. *fumigatus*	3562	/	1.15 ±0.18	1.42 ±.0.06	**17.19 ±2.01**	1.11 ±0.12	1.13 ±0.18	1.16 ±0.17	1.13 ±0.18	1.31 ±1.86
*C*. *cladosporioides*	859	/	1.02 ±0.16	2.01 ±0.34	1.45 ±0.06	**3.52 ±0.05**	1.50 ±0.07	1.32 ±0.15	1.69 ±0.69	1.45 ±0.20
*C*. *herbarum*	2268	/	2.19 ±0.70	2.94 ±0.06	1.29 ±0.89	1.98 ±0.89	**4.02 ±1.35**	1.63 ±0.35	1.67 ±0.75	2.36 ±0.32
*P*. *chrysogenum*	20859	/	1.04 ±0.23	2.95 ±0.87	0.87±0.03	1.03 ±0.18	0.99 ±0.13	**10.69 ±4.14**	1.16 ±0.23	0.88 ±0.07
*S*. *chartarum*	359	/	1.22 ±0.16	0.94 ±0.22	1.21 ±0.22	1.33 ±0.27	1.23 ±0.19	1.28 ±0.33	**21.59 ±0.20**	1.14 ±0.87
*U*. *botrytis*	328	/	2.03 ±0.99	1.05 ±0.07	1.26 ±0.14	1.44 ±0.02	2.00 ±0.13	1.38 ± 0.00	1.43 ±0.18	**27.15 ±0.18**

^a^ Identification number as defined by the BCCM/IHEM collection, Mycology and Aerobiology, Scientific Institute for Public Health, Juliette Wytsman street 14, 1050 Brussels, Belgium.

^b^ NTC defined as no template control.

^c^ Positive results obtained with 3 independents runs of independent gDNA extracts of pure cultures run in duplicate. A result is considered positive when the average (±standard deviation) of the SN ratio is ≥3.00.

^d^ In bold are the values considered as positive (i.e., average SN ratio ≥3.00).

#### DNA mix analysis

Subsequently, an analysis was performed on 9 different DNA mixes (Table 2), with a design allowing to verify that the detection of each species is still possible in the presence of other species and with a mix of set of beads. All the positive controls were correctly detected for each repetition (6/6) with a SN ratio ranging between 4.31 ±0.92 for *A*. *fumigatus* and 17.82 ±0.63 for *A*. *versicolor*. The first mix contained all of the targeted species. The Luminex analysis resulted in an SN ratio ≥3.00 (i.e. all positive) for all of the expected species and probes for each repetition (6/6 positive for all), with a lowest SN ratio observed at 4.69 ±0.68 (for *A*. *fumigatus*) and the highest SN ratio observed at 16.98 ±2.85 (for *A*. *versicolor*) (Table 5). In the other mixes (mixes 2 to 9) each time one species was omitted (Tables 2 and 5). The gDNA of each target species introduced in the mixes was each time detected (6/6) with SN ratios ranging from 3.37 ±0.25 for the detection of *S*. *chartarum* in the mix 7 to 26.10 ±1.37 for *P*. *chrysogenum* in the mix 4 (Table 5), indicating that no false negatives were obtained. No positive Luminex signal was obtained for any coupled bead sets for which no corresponding specific gDNA was added to the mix (SN ratios ranged between 0.92 ±0.20 and 2.62 ±0.25) (Table 5). This means that for the 6 repetitions, no false positives were observed during this test.

**Table 5:** DNA Mixes analysis.

Species	BCCM/IHEM strain ^a^	NTC ^b^	Number of positives ^c^									
			Positive control ^e^	Mix 1	Mix 2	Mix 3	Mix 4	Mix 5	Mix 6	Mix 7	Mix 8	Mix 9
*A*.*alternata*	3320	0/6	6/6	6/6	**0/6**	6/6	6/6	6/6	6/6	6/6	6/6	6/6
*A*. *versicolor*^*g*^	18884	0/6	6/6	6/6	6/6	**0/6**	6/6	6/6	6/6	6/6	6/6	6/6
*A*. *fumigatus*	3562	0/6	6/6	6/6	6/6	6/6	**0/6**	6/6	6/6	6/6	6/6	6/6
*C*. *cladosporioides*	859	0/6	6/6	6/6	6/6	6/6	6/6	**0/6**	6/6	6/6	6/6	6/6
*C*. *herbarum*	2268	0/6	6/6	6/6	6/6	6/6	6/6	6/6	**0/6**	6/6	6/6	6/6
*P*. *chrysogenum*	20859	0/6	6/6	6/6	6/6	6/6	6/6	6/6	6/6	**0/6**	6/6	6/6
*S*. *chartarum*	359	0/6	6/6	6/6	6/6	6/6	6/6	6/6	6/6	6/6	**0/6**	6/6
*U*. *botrytis*	328	0/6	6/6	6/6	6/6	6/6	6/6	6/6	6/6	6/6	6/6	**0/6**
Species	BCCM/IHEM strain ^a^	NTC ^b^	SN ratio ± SD ^d^^,^^e^									
			Positive control ^e^	Mix 1	Mix 2	Mix 3	Mix 4	Mix 5	Mix 6	Mix 7	Mix 8	Mix 9
*A*.*alternata*	3320	ND ^f^	6.98 ±0.83	7.73 ±1.95	**1.20 ±0.35**	5.75 ±1.36	6.18 ±1.17	8.49 ±0.14	5.39 ±0.33	6.82 ±0.89	4.90 ±0.17	5.40 ±0.32
*A*. *versicolor*^*g*^	18884	ND	17.82 ±0.63	16.98 ±2.85	13.88 ±0.04	**1.23 ±0.03**	18.83 ±0.37	18.29 ±0.06	17.34 ±0.29	10.93 ±0.54	17.35 ±1.31	15.52 ±0.57
*A*. *fumigatus*	3562	ND	4.31 ±0.92*	4.69 ±0.68	4.27 ±0.09	4.27 ±0.19	**2.43 ±0.33**	5.08 ±1.67*	4.06 ±0.63*	3.47 ±0.34*	4.58 ±0.17	5.85 ±1.02
*C*. *cladosporioides*	859	ND	10.04 ±0.74	5.40 ±0.15	5.52 ±1.65	10.08 ± 2.49	3.81 ±0.69*	**1.81 ±0.63**	5.39 ±0.46	8.72 ±1.03	5.85 ±1.69	7.33 ±0.96
*C*. *herbarum*	2268	ND	6.39 ±0.23	6.56 ±0.25	5.39 ±1.23	5.26 ±0.09	20.55 ±0.63	8.72 ±0.26	**2.62 ±0.25**	5.98 ±1.79	4.12 ±0.23	7.33 ±1.23
*P*. *chrysogenum*	20859	ND	6.66 ±1.26	7.69 ±1.48	4.39 ±0.02	4.07 ± 0.27*	26.10 ±1.37	9.01 ±1.97	4.85 ±0.26	**0.92 ±0.20**	9.68 ±1.25	8.41±2.01
*S*. *chartarum*	359	ND	7.04 ±0.75	6.54 ±1.53	5.87 ±0.01	5.87 ±1.21	4.41 ±0.82*	6.16 ±1.30	4.46 ±0.45	3.37 ±0.25*	**1.77 ±0.41**	4.42 ±0.10
*U*. *botrytis*	328	ND	8.90 ±1.02	8.16 ±1.90	8.31 ±0.21	7.32 ±1.62	6.02 ±0.54	9.18 ±0.19	5.31 ±0.64	7.53 ±1.38	5.06 ±0.29	**1.81 ±0.05**

^a^ Identification number as defined by the BCCM/IHEM collection, Mycology and Aerobiology, Scientific Institute for Public Health, Juliette Wytsman street 14, 1050 Brussels, Belgium.

^b^ No Template control.

^c^ Number of positive detections obtained during 3 independent runs done in duplicate.

^d^ SN ratio defined as the average (± standard deviation) of the ratio between the MFI values for the sample and the NTC for a specific target, obtained with 3 independents runs of independent gDNA extracts of pure cultures (5 ng of gDNA).

* indicates the significance of each result obtained during a t-test (confidence 95%) performed between the SN ratios obtained for the mix and the negative control mix of the targeted species (in bold).

^e^ In bold are the values considered as negative (i.e., average SN ratio <3.00).

^f^ ND defined as not detected.

^g^ Only DNA of *A*. *versicolor* was added for this assay. The probe VersP1 is not specific and detects also the closely related species *A*. *creber* and *A*. *sydowii*. *A*. *versicolor* is however more commonly observed in indoor air than the 2 other species.

### Sensitivity: Limit of detection

The sensitivity of this Luminex assay was determined with 9 points of serial dilutions (from 10 to 0.001 ng of gDNA for each of the targeted species). The LOD was 0.05 ng for *A*. *alternata*, *A*. *creber*, *A*. *sydowii*, *A*. *fumigatus*, *C*. *herbarum*, *P*. *chrysogenum* and *S*. *chartarum* and 0.01 ng for *A*. *versicolor*, *C*. *cladosporioides* and *U*. *botrytis* (Table 6).

**Table 6:** Limit of detection ^a^ of the fungal Luminex assay.

Species	DNA amount (ng)									LOD (ng)
	10	5	1	0.5	0.1	0.05	0.01	0.005	0.001	
*A*. *alternata*	100^b^ (6/6)	100 (6/6)	100 (6/6)	100 (6/6)	100 (6/6)	**100 (6/6)**	83 (5/6)	50 (3/6)	0 (0/6)	0.05
*A*. *creber*	100 (6/6)	100 (6/6)	100 (6/6)	100 (6/6)	100 (6/6)	**100 (6/6)**	83 (5/6)	50 (3/6)	0 (0/6)	0.05
*A*. *sydowii*	100 (6/6)	100 (6/6)	100 (6/6)	100 (6/6)	100 (6/6)	**100 (6/6)**	67 (4/6)	50 (3/6)	0 (0/6)	0.05
*A*. *versicolor*	100 (6/6)	100 (6/6)	100 (6/6)	100 (6/6)	100 (6/6)	100 (6/6)	**100 (6/6)**	83 (5/6)	33 (2/6)	0.01
*A*. *fumigatus*	100 (6/6)	100 (6/6)	100 (6/6)	100 (6/6)	100 (6/6)	**100 (6/6)**	50 (3/6)	33 (2/6)	0 (0/6)	0.05
*C*. *cladosporioides*	100 (6/6)	100 (6/6)	100 (6/6)	100 (6/6)	100 (6/6)	100 (6/6)	**100 (6/6)**	83 (5/6)	17 (1/6)	0.01
*C*. *herbarum*	100 (6/6)	100 (6/6)	100 (6/6)	100 (6/6)	100 (6/6)	**100 (6/6)**	50 (3/6)	50 (3/6)	17 (1/6)	0.05
*P*. *chrysogenum*	100 (6/6)	100 (6/6)	100 (6/6)	100 (6/6)	100 (6/6)	**100 (6/6)**	83 (5/6)	17 (1/6)	0 (0/6)	0.05
*S*. *chartarum*	100 (6/6)	100 (6/6)	100 (6/6)	100 (6/6)	100 (6/6)	**100 (6/6)**	67 (4/6)	67 (4/6)	50 (3/6)	0.05
*U*. *botrytis*	100 (6/6)	100 (6/6)	100 (6/6)	100 (6/6)	100 (6/6)	100 (6/6)	**100 (6/6)**	50 (3/6)	17 (1//6)	0.01

^a^ Limit of detection (LOD) obtained in duplicate with 3 independents runs of independent gDNA extracts of pure cultures. In bold: results defining the LOD.

^b^ The % of positive results. The number of positive results is indicated between brackets.

### Proof of concept using environmental samples

Following the performance assessment, DNA extracted from environmental indoor air samples collected from different rooms inside a contaminated house was analyzed using the fungal Luminex assay. This gave the opportunity to test the molecular tool with real-life samples containing a mix of a priori unknown fungal species at unknown concentration. Therefore, in this study, the proof of concept using environmental samples allows not only testing the performance (detection of DNA at very low or variable concentrations, detection in a mix of species, etc.) of the developed tool but it allows also demonstrating that the developed tool can be used with uncharacterized strains (i.e., strains not coming from a culture collection) of targeted species which could be present in an environmental air sample, and this using DNA extracted from the air samples without prior cultivation step.

The 4 samples used in this study were previously analyzed with classical methods [10] and only 3 species were determined i.e., *A*. *versicolor*, *Cladosporium* sp. and *P*. *chrysogenum*. *P*. *chrysogenum* was observed in each sample, while *A*. *versicolor* was retrieved in 2 of them (i.e., samples from bathroom and living room) and *C*. *cladosporioides* in sample 4 (i.e., sample collected in the bathroom) only. Infertile mycelia were also observed in the living room and the kitchen (Table 7).

**Table 7:** Proof of concept with environmental samples: Culture, microscopic determination and quantification.

Sampling place	Species	Number of colonies on plate	CFU/m^3^ ^a^
Bedroom	infertile mycelium	4	50
	*P*. *chrysogenum*	17	213
Kitchen	*P*. *chrysogenum*	6	75
	infertile mycelium	3	38
Living room	*A*. *versicolor*	1	13
	*P*. *chrysogenum*	18	225
Bathroom	*A*. *versicolor*	1	13
	*Cladosporium* sp.	1	13
	*P*. *chrysogenum*	15	188

^a^ The value for CFU/m^3^ is an estimation of fungal contamination based on the number of colonies per plate.

These 3 species were also detected in the same rooms by the fungal Luminex assay performed on the extracted DNA from these 4 real-life samples (Table 8). SN ratios obtained for each species were 3.84 ± 0.18 for *C*. *cladosporioides*, 3.67 ±0.04 and 3.34 ±0.05 for *A*. *versicolor*, respectively in the bathroom and the living room and ranged between 3.77 ± 0.09 in the kitchen and 14.83 ±0.22 for *P*. *chrysogenum* in the bathroom (Table 8). No other species were detected (Table 8). Because the probe VersP1 is not specific to *A*. *versicolor* (Tables 1, 2, 3 and 4), it is important to note that the taxa detected in the sample could also be *A*. *creber* and *A*. *sydowii*. However, according to the microscopic visualization and based on the fact that *A*. *versicolor* is more frequently observed in indoor air from Belgium than *A*. *creber* and *A*. *sydowii*, the detected species was considered as *A*. *versicolor* (Table 8).

**Table 8:** Proof of concept with environmental samples: Luminex xMAP^®^ analysis.

Species	Control ^a^^,^^b^^,^^d^	Environmental sample ^d^				Spike test ^c^^,^ ^d^			
		1. Bedroom	2.Kitchen	3.Bathroom	4.Living room	Spike 1(bedroom)	Spike 2 (kitchen)	Spike 3 (bathroom)	Spike 4 (living room)
*A*. *alternata*	4.16 ±0.68*	0.72 ±0.17	0.98 ±0.53	1.41 ±0.09	1.12 ±0.01	3.74 ±0.10*	4.29 ±0.09*	3.26 ±0.26*	4.03 ±0.57*
*A*. *versicolor* ^*e*^	**4.22 ±0.22**	0.94 ±0.16	1.91 ±0.53	3.67 ±0.04*	3.34 ±0.05*	4.91 ±0.55*	4.09 ±0.71*	*3*.*78 ±0*.27*	*3*.*18 ±0*.12*
*A*. *fumigatus*	3.41 ±0.14*	2.03 ±0.62	2.16 ±0.35	1.39 ±0.03	1.72 ±0.49	4.18 ±0.52*	3.69 ±0.21*	**3.08 ±0.35** *	3.05 ±0.39*
*C*. *cladosporioides*	3.44 ±0.04*	0.72 ±0.20	0.76 ±0.25	0.88 ±0.08	3.84 ±0.18*	3.57 ±0.16*	3.58 ±0.17*	3.60 ±0.14*	*3*.*23 ±1*.02*
*C*. *herbarum*	3.01 ±0.06*	0.62 ±0.17	0.71 ±0.22	1.59 ±0.62	1.11 ±0.01	4.12 ±0.76*	3.93 ±0.50*	4.10 ±0.28*	3.83 ±0.15*
*P*. *chrysogenum*	3.00 ±0.09*	**7.77 ±0.22**	**3.77 ±0.09**	**14.83 ±0.22**	**12.27 ±0.23**	***9*.*40 ±2*.*46***	***3*.*60 ±0*.*11***	***3*.*64 ±0*.*01***	***4*.*47 ±1*.*39***
*S*. *chartarum*	**4.82 ±0.02**	0.64 ±0.16	0.60 ±0.16	1.54 ±0.16	1.43 ±0.04	9.41 ±2.46*	7.09 ±0.07*	4.81 ±1.51*	3.99 ±0.66*
*U*. *botrytis*	8.68 ±0.52*	0.60 ±0.13	0.36 ±0.13	1.05 ±0.17	1.24 ±0.48	6.15 ±0.38*	4.92 ±1.13*	4.61 ±0.70*	3.70 ±0.99*

^a^ BCCM/IHEM 3320 for *A*. *alternata*, BCCM/IHEM 18884 for *A*. *versicolor*, BCCM/IHEM 3562 for *A*. *fumigatus*, BCMM/IHEM 859 for *C*. *cladosporioides*, BCCM/IHEM 2268 for *C*. *herbarum*, BCCM/IHEM 20859 for *P*. *chrysogenum*, BCCM/IHEM 359 for *S*. *chartarum* and BCCM/IHEM 328 for *U*. *botrytis*. BCCM/IHEM collection, Mycology and Aerobiology, Scientific Institute for Public Health, Juliette Wytsman street 14, 1050 Brussels, Belgium.

^b^ SN ratio defined as the average (± standard deviation) of the ratio between the MFI values for the sample and the NTC for a specific target, obtained with 3 independents runs of independent gDNA extracts of pure cultures (5 ng of gDNA). In bold are the values considered as positive (i.e., average SN ratio ≥3.00).

* indicates the significance of each SN ratio close to the limit (i.e. 3) was evaluated with t-test (confidence 95%) performed with the higher negative results considered as a worst negative control.

^c^ In each air sample, 5 ng DNA extracted from each strain not detected in the indoor samples were spiked into the DNA extracted from the air samples. The SN ratios of each species detected in air samples (not spiked) were put in italic. DNA used for the spike comes from the same strains than those used as positive control.

^d^ In bold are the values considered as positive (i.e., average SN ratio ≥3.00).

^e^ Because the VersP1 probe is not specific to *A*. *versicolor*, the species detected could also be *A*. *sydowii* or *A*. *creber*, except for those where a spike with *A*. *versicolor* was made.

In order to verify that no inhibition occurred during the analysis of the environmental samples, and to verify that if a species would have been present, it could be detected in the 4 environmental samples, a spike of all species not detected by the Luminex assay was performed into every environmental sample, according to the results obtained for the first part of the proof of concept analysis. Therefore, based on the results obtained for the analysis of the 4 environmental samples, *A*. *versicolor* was spiked into the samples 1 and 2, but not into samples 3 and 4. *C*. *cladosporioides* was spiked into the samples 1 to 3 and not in sample 4. Finally, as it was detected in each sample, no spike of *P*. *chrysogenum* was performed. Every gDNA spiked into the 4 samples was properly detected (Table 8). Indeed, in the 4 samples each species, spiked or not (as already present), was detected (SN ratio ≥3.00).

## Discussion

For years, fungal indoor air contamination is considered as a public health problem, even if today no substantiated scientific evidence on the causal link exists. This lack of evidence is principally due to a scarcity of data on the full composition of indoor airborne fungal community. Indeed, most of the protocols used today for fungal contamination monitoring are culture-dependent. This implies some limitations, such as that they are not able to reflect the complete indoor fungal community representativeness, as the dead and uncultivable fraction is not detected with these methods. Moreover, they are time-consuming. To improve the collection of data in terms of time and completeness, molecular methods, such as qPCR, have been developed for the detection and identification of the indoor fungal community. However, even if the efficiency of most of these qPCR methods has been well established, their multiplex capacities are still too limited. With its ability to simultaneously analyze up to 50 different targets, the use of the Luminex xMAP^®^ technology on a MagPix instrument could significantly upgrade the indoor fungal contamination monitoring as was previously demonstrated for the diagnosis of some relevant fungal and other pathogens from clinical samples [20, 21, 26, 32–34].

This study presents the first Luminex xMAP^®^ assay developed for the monitoring of 10 fungal species most frequently found in indoor air in Europe and that may cause health problems, i.e., *A*. *alternata*, *A*. *creber*, *A*. *fumigatus*, *A*. *sydowii*, *A*. *versicolor*, *C*. *cladosporioides*, *C*. *herbarum*, *P*. *chrysogenum*, *S*. *chartarum* and *U*. *botrytis* [2, 22, 23]. While *A*. *creber*, *A*. *versicolor*, *A*. *sydowii* and *P*. *chrysogenum* are typical indoor species, *A*. *alternata*, *A*. *fumigatus* and *C*. *herbarum* are known to be outdoor species. Despite their outdoor sources, these 3 species are commonly detected in indoor air samples. Especially observed in the forms of spores, they arrive in indoor environment through the draft and ventilation system (i.e., windows, ventilation or air-conditioning system).

Some of these species such as *A*. *alternata*, *A*. *versicolor*, *P*. *chrysogenum*, *U*. *botrytis* or *S*. *chartarum* are known to have implications in the worsening of respiratory diseases or allergies [5, 35–39]. The detection of these multiple species is therefore important in the context of health issues. The Luminex xMAP^®^ technology gives the opportunity to perform in a single run a multiplex analysis able to detect multiple species with high specificity. This specificity is itself defined by the specificity of the probes bound to the each set of beads. These beads are the key elements of this technology.

To have a species-specific detection, the probes were designed to target the ITS1 or ITS2 regions from the ribosomal DNA, which are recognized as the most suitable region for the detection of fungi due to their low intra-species variablility [40, 41]. There exist universal primer pairs (i.e. ITS1/2 and ITS 3/4 [24]) to amplify these regions in all fungi. Therefore, targeting of ITS1 and ITS2 also allowed to easily design a multiplex detection based on species-specific probes. Indeed, the targeting of the ITS regions and the use of one set of universal primers for each region allow to reduce the number of step. Consequently, the time and amount of sample needed for the generation of a species-specific amplicon were also decreased. Actually, the PCR amplifications could be done in a duplex PCR reaction with universal primers for fungal gDNA amplification (primers ITS1, 2, 3 and 4). Due to their universal character, the use of these primers also gives the possibility to extend the panel of species to be detected in the future, by adding specific probe-coupled bead sets. This will even more improve the powerful fungal monitoring based on this xMAP^®^ assay. Indeed, the specific detection of the generated amplicons is based on the hybridization of these amplicons to specific probes coupled to Luminex bead-sets. Every probe used in this study originates from a previously developed TaqMan^®^ qPCR method (Table 1) [17, 20]. Therefore, the specificity of each probe used in this study was previously validated [17, 20]. According to the Luminex’ recommandations, the optimal probe length used for an xMAP^®^ analysis is defined between 15 and 20 nucleotides [19]. However, all probes used in this study, obtained from literature, are larger than 20 nucleotides. For most of them, according to the *in silico* analysis, their specificity was negatively impacted by a size reduction. Therefore, in this study the length defined for the best detection of each species targeted in our mulitplex assays was found to be around 30 nucleotides. To optimize the TMAC-based hybridization step, which is probe-size dependent, all the probes were adapted to have a length between 28 and 31 nuclotides.

The current assay showed a specific detection of each targeted species, as observed during the simplex and multiplex assays. The species-specific detection was further demonstrated with the DNA mixes test. No false negative nor false positives were observed. The remark has to be made that the developed fungal Luminex assay targets 10 different fungal species. Seven of them are detected uniquely with specific probes. The 3 remaining closely related species (i.e., *A*. *creber*, *A*. *sydowii* and *A*. *versicolor*) are detected by one and the same probe, VersP1. This implicates that although the 3 species can be detected using the Luminex assay, no discrimination can be made between them. The poor discrimination between these 3 *Versicolores* (*A*. *creber*, *A*. *sydowii* and *A*. *versicolor*) species was already observed in other studies [10, 15]. To improve the specific identification of these 3 species, an additional marker could be added such as the gene coding for β-tubulin or mycotoxin genes [20, 42]. However, the use of a new marker requires the optimization of the PCR workflow (multiplex optimization or addition of an amplification step) and probably of the hybridization temperature in the xMAP^®^ workflow, which depends on the probe length. Indeed, with the use of TMAC, the specificity of the annealing depends only on the hybridization temperature, and not on the composition of the probe. Therefore, to avoid any problem of specificity, adding markers (and thus probes) to a workflow will require the optimization of this parameter. When using multiple markers to target closely related species such as the 3 *Versicolores* in this study, a decision-tree-based workflow could be applied after the Luminex assay to perform the discrimination based on the combination of the signals obtained for each marker. With such a decision-tree-based workflow, the xMAP^®^ assay developed in this study could already be used as a first screening without modifications. If a positive result is observed for the VersP1 probe, a second analysis could be performed with some new markers in order to identify the species. This kind of decision tree already exists in other fields e.g., for the detection of genetically modified plants [43]. Alternatively, another molecuar assay could be used, e.g. based on high resolution melting qPCR for the discrimination of closely related species [44]. Being able to discriminate between these 3 species will contribute to our understanding of the impact of indoor fungal contamination and health problems, as currently, the presence of these 3 *Aspergillus* species in indoor air and their difference in impact on health could not yet be evaluated.

In addition to its specificity, the xMAP^®^ assay developed in this study is also sensitive with a LOD ranging between 0.05 and 0.01 ng of gDNA. According to the limited information available on the genome size of each targeted species, these LOD correspond to a range from 2.51 theoretical genomic copy numbers for *C*. *cladosporioides* and *U*. *botrytis*, to 19.84 theoretical genomic copy numbers for *A*. *fumigatus* (Table 9). It should be noted that intraspecies ITS copy number variations observed in some fungal species such as *A*. *alternata*, *A*. *fumigatus*, *A*. *versicolor* or *C*. *cladosporioides* could affect the LOD [10, 13, 45, 46], thereby impeding the theoretical genomic copy number variation estimation. Therefore, it has been decided to determine the LOD in mass, instead of in genomic copy numbers.

**Table 9:** Theoretical genomic copy number estimation of the LOD.

Species	Genome size (Mb)	Reference	LOD	
			DNA amout (ng)	CN estimation ^a^
*A*. *alternata*	32.99	[47]	0.05	14.06
*A*. *creber*	33.76 ^b^	[47]	0.05	13.74
*A*. *fumigatus*	29.39	[47]	0.05	19.84
*A*. *sydowii*	34.38	[47]	0.05	13.49
*A*. *versicolor*	33.13	[47]	0.01	2.80
*C*. *cladosporioides*	36.91^c^	[48]	0.01	2.51
*C*. *herbarum*	36.91 ^c^	[48]	0.05	12.57
*P*. *chrysogenum*	31.34	[47]	0.05	14.80
*S*. *chartarum*	40 ^d^	[49]	0.05	11.60
*U*. *botrytis*	36.91 ^c^	[48]	0.01	2.51

^a^ CN estimation defined as an estimation of the theoretical gDNA copy number (CN).

^b^ No sequencing data available; genome size obtained as an average of the genome size from the other species from the Versicolores group i.e., *A*. *sydowii* and *A*. *versicolor*.

^c^ No sequencing data available; genome size considered as general estimation of the Ascomycota genome size.

^d^ Genome size estimation based on the whole genome sequencing of the environmental strain *S*. *chartarum* 51–11.

The proof of concept using environmental samples showed that the xMAP^®^ technology can be used for the fungal monitoring in indoor environment. Indeed, the 3 species detected with the classical methods (i.e., *A*. *versicolor*, *C*. *cladosporioides* and *P*. *chrysogenum*) were also detected with the xMAP^®^ technology and this without prior cultivation of the sample. Also, the species not detected with the classical protocol were not detected with the xMAP^®^ assay. This test indicates that our Luminex assay can be used on mixed and unbalanced concentrations of fungal species without giving the problem of false detection. This observation is supported by the spike test results which showed that the species present in the gDNA extracted from the environmental samples (not spiked) could still be detected even when a high amount of spiked DNA of other species was present. The spike test results also demonstrated that no inhibitors were present in the DNA mix, as the spiked DNA could be detected by the Luminex assay performed on the environmental samples. If the species would have been present in the environmental sample, it would have been detected by the Luminex assay. So, if the species was not detected, it was not present in the environmental samples, or it was present at a concentration below the LOD. According to the observations based on culturing, besides *P*. *chrysogenum*, also some infertile mycelia were observed with classical analysis in samples from the kitchen and the bedroom while the xMAP^®^ assay only detected *P*. *chrysogenum* (Tables 7 and 8). According to these results and those from the spike test, these undetermined taxa do not belong to the targeted species, except for *P*. *chrysogenum*. As no DNA sequencing was performed on the infertile mycelia observed on plate, we cannot exclude that these infertile mycelia belong to an untargeted species of our xMAP^®^ assay.

However, if needed, probes for additional taxa, once determined, can be easily added to the assay, as elaborated above. Additionally, a ‘general ITS’ probe, detecting all fungal species, could be added to the assay. If none of the specific probes give a Luminex signal, but there is fungal DNA present in the sample (as detected by the general probe), other methodologies, including mass parallel sequencing, could be applied to further characterize the sample, if needed. Nevertheless, this environmental test delivered the proof of concept for the use of the xMAP^®^ technology, which is culture-independent and less time-consuming for the analysis of real-life samples i.e., 3 days for the xMAP^®^ technology (sampling, DNA extraction included) compared to 5 to 21 days for the classical analysis (sampling and the culturing included).

Our fungal assay has been developed as a detection tool. Because the Luminex xMAP^®^ method contains at least one step of PCR amplification, the tool developed in this study should not be considered as a quantitative one. Indeed, due to the exponential amplification of the DNA, the quantification cannot be done accurately, i.e. the detected fluorescence does not translate directly to the number of DNA molecules in the sample. It can merely be considered as a semi-quantitative tool. Additionally, there exists a species- and even strain-dependent copy number variation of the ITS marker, which is still poorly documented as previously discussed [10]. This also complicated the quantification based on this marker. To develop quantitative tools, more studies would have to be made to find a marker that is very well conserved all along the fungal kingdom, and with a constant copy number, preferably a single copy gene, and which could be used for the quantification. Hereto, there is a need for more whole genome sequence data of fungal species (and this for multiple strains per species) to screen the fungal genome in order to find a conserved single copy marker or a unique single copy marker per species, as was previously found for *A*. *fumigatus* [45]. Another solution could also be found in the use of new technologies such as digital PCR, which allows absolute quantification, as was done for *Candida albicans* in blood [50]. However, as digital PCR requires an *a priori* selection of the fungal species, an absolute quantification of each contaminant cannot be made without a prior screening of the diversity. This screening step, performed before the quantification step, could be done using the Luminex xMAP^®^ fungal assay or a metagenomics NGS analysis.

To conclude, this study reported on a fast, specific and sensitive Luminex xMAP^®^ assay targeting 10 important fungal contaminants frequently observed in indoor air and that could have health impacts. The use of the xMAP^®^ technology allows a culture–independent analysis with a reduced turn-around-time compared to the classical protocols. The use of this molecular multiplex tool to investigate the indoor contamination could improve the monitoring of fungal diversity. The improvement of data on the fungal population in buildings will contribute to the knowledge concerning their impact on public health, especially on respiratory diseases.

## Ethical approval

This article does not contain any studies with human participants or animals performed by any of the authors.
